# Assembly and comparative analysis of the complete mitochondrial genome of red raspberry (*Rubus idaeus* L.) revealing repeat-mediated recombination and gene transfer

**DOI:** 10.1186/s12870-024-05969-7

**Published:** 2025-01-22

**Authors:** Huajie Zhang, Minghui Yan, Lijuan Li, Zhuo Jiang, Ye Xiong, Yusheng Wang, Caleb Onoja Akogwu, Olutayo Mary Tolulope, Hao Zhou, Yanxia Sun, Hengchang Wang

**Affiliations:** 1 CAS Key Laboratory of Plant Germplasm Enhancement and Specialty Agriculture, Wuhan Botanical Garden, Chinese Academy of Sciences, Wuhan, Hubei, 430074 China; 2 Center of Conservation Biology, Core Botanical Gardens, Chinese Academy of Sciences, Wuhan, Hubei, 430074 China; 3 University of Chinese Academy of Sciences, Beijing, 100049 China; 4 Dabie Mountain Laboratory, College of Tea and Food Science, Xinyang Normal University, Xinyang, Henan, 464000 China; 5 School of Horticulture and Gardening, Yangtze University, Jingzhou, Hubei, 434025 China; 6 Key Laboratory of Molecular Biophysics of the Ministry of Education, College of Life Science and Technology, Huazhong University of Science and Technology, Wuhan 430074, Hubei, China

**Keywords:** De-novo assembly, Mitogenome, Phylogenetic analysis, Recombination, Sequence collinearity

## Abstract

**Background:**

Red raspberry (*Rubus idaeus* L.) is a renowned fruit plant with significant medicinal value. Its nuclear genome and chloroplast genome (plastome) have been reported, while there is a lack of genetic information on its mitogenome. We sequenced and assembled the complete mitogenome of *R. idaeus*, and conducted a series of genetic investigations comparing it with the nuclear and chloroplast genomes, so as to better gain a comprehensive understanding of the species’ genetic background.

**Results:**

The mitogenome is represented by one circular chromosome of 438,947 bp. Twenty-four core genes, nine variable genes, 26 tRNAs, and three rRNAs were annotated. A total of 52 SSRs and 38 tandem repeat sequences were identified. 533 pairs of dispersed repeats were detected, among which three pairs were found to have mediated the homologous recombination, resulting in one major conformation and seven minor conformations. Furthermore, 52 homologous sequences between the mitogenome and plastome were identified, including six complete protein-coding genes and 12 tRNA genes. We also detected 828 homologous fragments between the nuclear genome and mitogenome, including one *trn*M-CAU gene.

**Conclusions:**

In this study, we presented the mitogenome of *R. idaeus* for the first time based on data obtained from Illumina and Oxford Nanopore sequencing platforms. Key characteristics of the mitogenome were examined, including its gene composition, repetitive elements, and homologous DNA fragments. Additionally, we identified multiple recombination events in the mitogenome mediated by repetitive sequences The high-quality and well-annotated mitogenome for the known fruit red raspberry will provide essential genetic information for species classification, evolution analysis, and even genetic improvement in *Rubus* in the future.

**Supplementary Information:**

The online version contains supplementary material available at 10.1186/s12870-024-05969-7.

## Background

*Rubus* L. (Rosaceae), comprising approximately 700 species, is a morphologically highly diversified group. The *Rubus* plants are broadly distributed in the world, with many species having edible wild fruits [1]. The most famous fruits are the red raspberry (*R. idaeus*) and black raspberry (*R. occidentalis*). Red raspberry is widely cultivated around the world, especially in the temperate zone of Europe [2, 3]. These fruits are rich in phenols, terpenoids, alkaloids, steroids, and fatty acids [4, 5]. The anthocyanins and other polyphenols in red raspberry have a range of potential anti-cancer and heart disease properties [6–8]. In China, the fruits of *R. idaeus* are known as "Fu pen zi” and are utilized in traditional Chinese medicine [9, 10]. Similarly, the leaves, roots, and stems have been reportedly used for medicinal purposes in Australia (diarrhea), China, and ancient Europe for wound healing and for difficulties during childbirth [10].

The genetic features of the cultivars studied may have a significant impact on both the yield and the qualitative characteristics of species [11]. Due to the economic importance, the whole genomes of red and black raspberry have been reported earlier [3, 12]. Relatively, in *Rubus*, the plastome data have been more widely used, e.g., for phylogenetic studies. Recently, the plastome of *R. idaeus* was published [13]. To date, plastomes of over 70 species have been completed [14–16]. Additionally, a study on the mitochondria of the Rosaceae has also been addressed [17]. Mitochondria are known as the "powerhouse" of cells that carry out oxidative metabolism and energy transformation [18]. Both mitochondria and chloroplast are semi-autonomous organelles, and their genetic system is relatively independent to the nucleus [19, 20]. Plant mitogenomes are powerful tools to study the origin of species, phylogenetics, and genetic diversity, for their genetic system is relatively independent of the nucleus and relatively informative [21–23]. They always exhibit a complex and dynamic structure. However, due to the complexity of plant mitogenome, e.g., extreme variation in genome structure and size, rich repetitive sequences, and the ability to incorporate foreign DNA [24–26], research into mitogenome always lag significantly, largely behind plastome.

Within the Rosaceae family, a total of 34 mitogenomes have been reported and analyzed in depth [17]. Their study revealed that repeats primarily drive the dynamics of genome structure through homologous recombination and genomic rearrangements [17]. However, in the genus *Rubus*, only one species’ mitogenome, that of *Rubus chingii*, has been reported to date. This limited information on mitogenomes is clearly insufficient for exploring the valuable germplasm resources of this popular fruit. In this study, we focused on investigating its mitogenome information and compared it with its other two sets of genomes, the nuclear and chloroplast genomes. Previous studies have shown that the wild accessions of red raspberries possess a higher antioxidant capacity, phytonutrient content, richer flavor, and more attractive color than the domesticated varieties [27]. Hence, we hope this study will provide significant genetic data for the comprehensive recognition of the fruit plant and potentially contribute to germplasm enhancement.

We assembled and annotated the mitogenome of wild *R. idaeus* and analyzed its gene content, repetitive sequences, repeat-mediated recombination, phylogenetic relationships, and codon preference. Furthermore, we identified the homologous DNA fragments between the mitogenome and plastome, as well as between the mitogenome and the nuclear genome. This study aims to analyze the complete mt genome of *R. idaeus* to provide insight for future studies on the genetic variations, phylogeny, and breeding of *R. idaeus*. Also, it provides an understanding of the conservation and utilization of medicinal economic plants.

## Results

### General feature of *R. idaeus* mitogenome

**Fig. 1:**
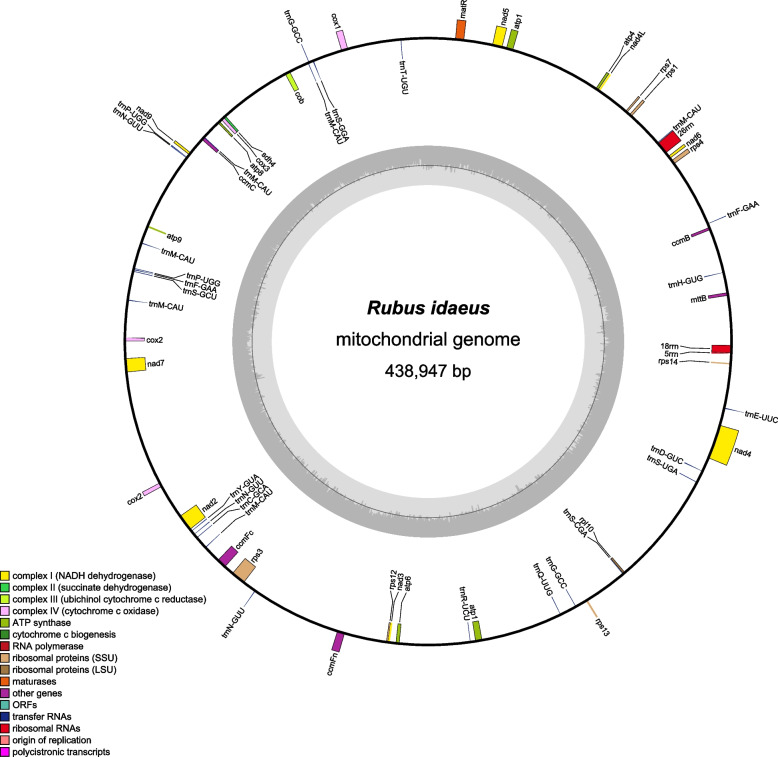
Mitogenome circular molecule of *R. idaeus*. The colored squares inside and outside the circle representing the various mitochondrial genes. Gene types with same function are marked with same colors

A total of 7 Gb of Illumina reads with a quality score greater than Q20, and 24 Gb of Nanopore reads with an N50 of 18,855 bp, were generated. These reads were assembled into a circular molecular structure (Fig. 1), resulting in a mitogenome length of 438,947 bp with a GC content of 44.1%. The base composition was A (27.91%), T (27.95%), C (22.03%) and G (22.11%). The intergenic regions accounted for 88.02% of the genome, indicating a relatively low gene density. A total of 33 protein-coding genes (PCGs) were annotated (Fig. 1; Table 1), including 24 core mitochondrial genes and nine variable genes. Additionally, 17 kinds of tRNA and three rRNA (*rrn*5, *rrn*18 and *rrn*26) were annotated. The core genes comprised five ATP synthase genes, nine NADH dehydrogenase genes, four cytochrome C biogenesis genes, three cytochrome C oxidase genes, one maturase (*mat*R), and one ubiquinol-cytochrome c reductase (*cob*) (Table 1). The variable genes included one large ribosomal protein subunit, eight small ribosomal protein subunits, and one succinate dehydrogenase gene (*sdh*4). Notably, the genes *atp*1 and *cox*2 were present in two copies. Among the 32 PCGs, seven genes contained introns, with three genes (*nad5*, *ccm*Fc and *rps*3) containing two introns each, two genes (*nad*2 and *nad*7) containing three introns each, and one gene (*nad*4) containing four introns (Fig. 1).

**Table 1:** Gene composition of the *R. idaeus *mitogenome

	Group of genes Name of genes	
Core genes	ATP synthase	*atp1*(2), *atp4*, *atp6*, *atp8*, *atp9*
	Complex I (NADH dehydrogenase)	*nad1, nad2*, *nad3*, *nad4*, *nad4L*, *nad5*, *nad6*, *nad7*, *nad9*
	Cytochrome c biogenesis	*cob*
	Ubiquinol cytochrome c reductase	*ccmB*, *ccmC*, *ccmFc*, *ccmFn*,
	Complex IV (cytochrome c oxidase)	*cox1*, *cox2*(2), *cox3*,
	Maturases	*matR*
	Transport membrane protein	*mttB*
Variable genes	Large subunit of ribosome	*rpl10*
	Small subunit of ribosome	*rps1*, *rps3*,* rps4*, *rps7*, *rps12*,* rps13*,* rps14*,
	Complex II (succinate dehydrogenase)	*sdh4*
rRNA genes	Ribosomal RNAs	*rrn5*, *rrn18*, *rrn26*
tRNA	Transfer RNAs	*trnC-GCA*, *trnD-GUC*, *trnE-UUC*, *trnF-GAA*(2,), *trnG-GCC*(2),*trnH-GUG*, *trnM-CAU*(6), *trnN-GUU*(2), *trnP-UGG*（2）, *trnQ-UUG*, *trnR-UCU*, *trnS-CGA*, *trnS-GCU* , *trnS-GGA*, *trnS-UGA*, *trnT-UGU* , *trnY-GUA*

### Codon usage analysis

**Fig. 2:**
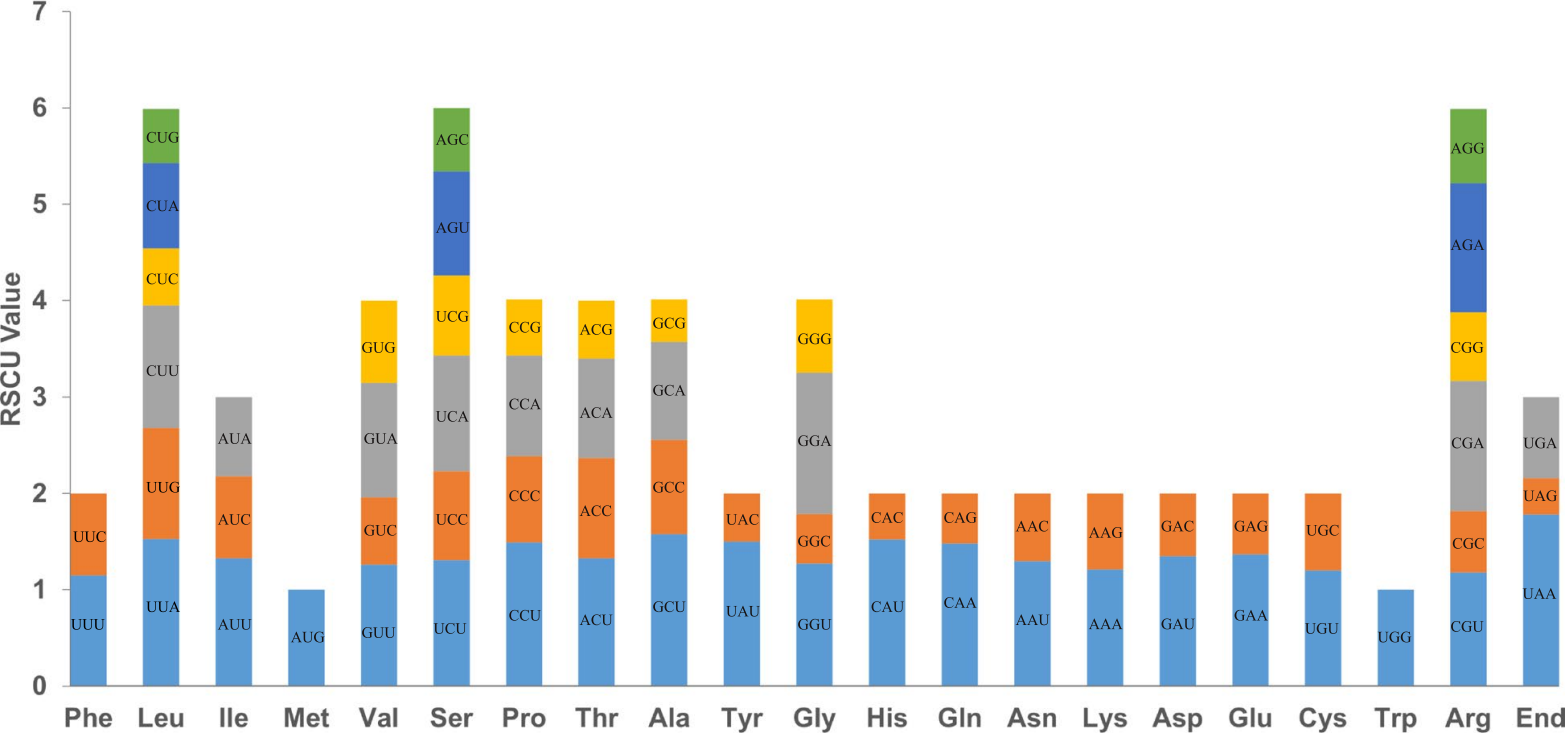
Codon preference of *Rubus idaeus* mitogenome

A codon usage analysis on the 33 PCGs of the *R. idaeus* mitogenome was conducted. Among these codons, leucine (Leu) was the most frequently used amino acid, followed by serine (Ser) and arginine (Arg). Relative synonymous codon usage (RSCU) values greater than 1 indicate a preference for specific codons for amino acids. Apart from the start codon AUG and tryptophan codon UGG, which both had an RSCU value of 1, the PCGs exhibited common codon usage preferences (Table S1; Fig. 2). The stop codon UAA showed the highest RSCU value of 1.78 among the PCGs, followed by alanine (Ala), which had a preference for the GCU codon with an RSCU value of 1.58. The RSCU values for UUA (leucine) and CAU (histidine) were also relatively high, with values of 1.53 and 1.52, respectively.

### Repeat elements

**Fig. 3:**
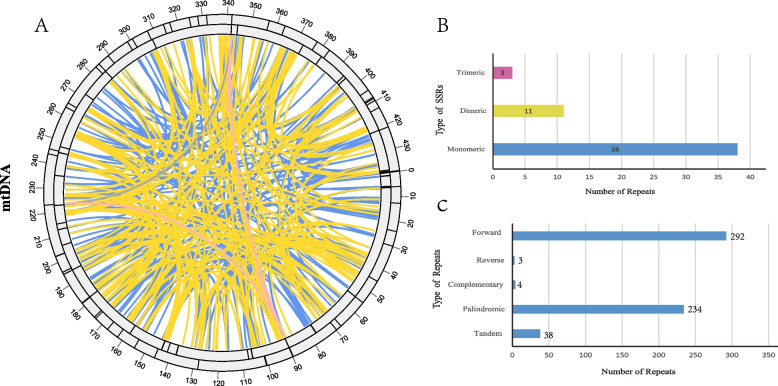
Repeat analysis of the mitogenome of *R. idaeus*. **A** The distribution of repetitive sequences. The inner circle showing the dispersed repeats connected with yellow and blue links. The next circles showing the tandem and microsatellite as short bars. The interval scale was 10 kb. **B** The distribution of different types of SSRs, the X axes representing the number of repeats, and the Y axes representing the types of repeats

A total of 52 SSRs were identified (Table S2; Fig. 3), with monomers and dimers comprising 94.23% of these repeats. Among the monomer SSRs, adenine (A) and thymine (T) repeats were the most common, accounting for 42.1% (No.16) and 44.74% (No.17) of the 38 monomer SSRs, respectively. Dimer SSRs included combinations such as AT, TA, CT, and AG. Notably, only two hexamer SSRs were observed, which included TTA, ATA, and CTT (Fig. 3). Additionally, 38 tandem repeat sequences were identified, each ranging in length from 9 to 54 bp (Table S3). There were also 533 pairs of dispersed repeats with lengths of 30 bp or greater, including 234 pairs of palindromic repeats, 292 pairs of forward repeats, three pairs of reverse repeats, and four pairs of complement repeats (Table S4; Fig. 3C). The number of dispersed repeats significantly exceeded that of SSRs and tandem repeats, with 32 dispersed repeats being longer than 100 bp. The longest palindromic repeat was 1,561 bp, located between positions 340,350 to 341,910 bp and 89,787 to 91,347 bp, and encompassed most of the *atp*1 gene sequence. The longest forward repeat was 461 bp, with repeat units positioned between 104,528 to 104,988 bp and 92,411 to 92,871 bp in the mitogenome (Fig. 3).

### Repeat-mediated homologous recombination

**Fig. 4:**
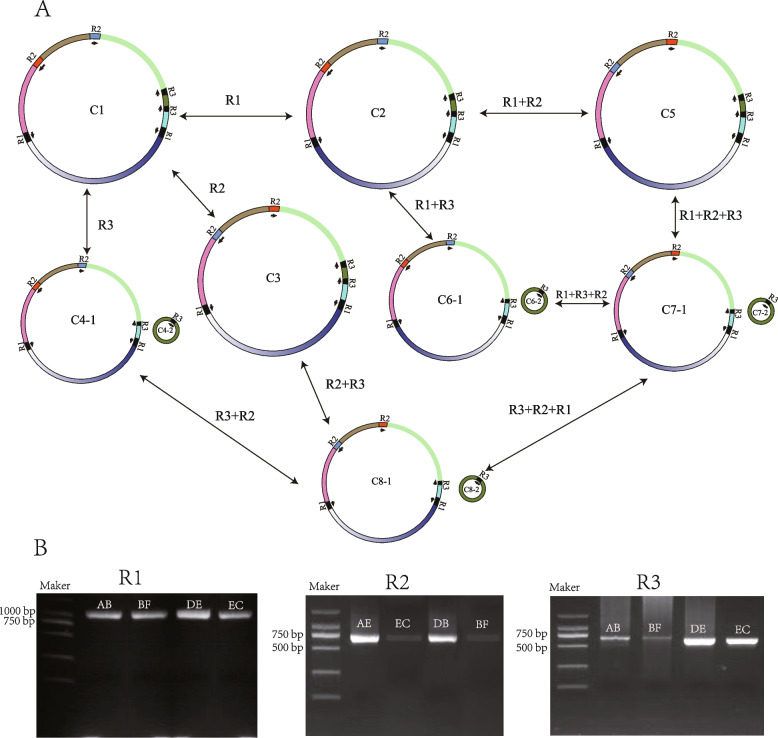
**A** Diverse mitochondrial genome combinations mediated by repeat sequences. C1 represents the assembled mitogenome. C2-C8 represents potential combinations mediated by three pairs of repeat sequences (R1, R2, and R3). The colored blocking within each circular ring. The black arrows indicate the direction of the repeat sequences. **B** The gel electrophoresis results of PCR products amplified using primers. The amplified sequences correspond to the four sites of the recombination conformation, including repeat sequence and flanking regions of 200 bp. The composition of the amplified sequences is represented by A-E, with different combination patterns corresponding to the recombination mechanisms in Fig S1

Three pairs of repeat sequences that may mediate homologous recombination were identified in the red raspberry mitogenome, validated using long-read sequencing. These sequences, designated as R1, R2, and R3, had lengths of 1,561 bp, 534 bp, and 461 bp, respectively. R1 and R2 were reverse repeats, while R3 was a forward repeat. R1 and R2 were 100% identical, with the inverted repeat R1 contributing to genome inversions. The inverted repeat R2 was linked to the formation of a single circular molecule, whereas the forward repeat R3 led to the generation of two independent circular molecules. The potential homologous recombination type in the red raspberry mitogenome was presented (Fig. 4A). The PCR experiment and Sanger sequencing were consistent with the expected sizes of the bands and correctness of the potential genomic configuration (Fig. 4B).

### Gene transfer within organelle genomes

**Fig. 5:**
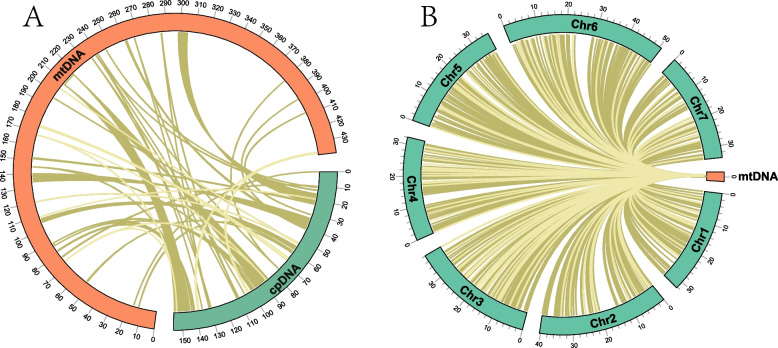
Homologous fragments analysis. **A** Homologous sequences between mitogenome and plastome of *R. idaeus*. The red arcs representing the mitogenome, and the green arcs, the plastome. **B** Homologous sequences between mitogenome and nuclear genome of *R. idaeus.* The green arcs showing the nuclear genome, the red arcs, the mitogenome

The size of the *R. idaeus* plastome was 158,638 bp [13]. According to sequence similarity, 52 DNA fragments homologous to the plastome were detected in the mitogenome (Fig. 5A and Table S5). The length of the homologous sequences is 46,456 bp, accounting for 10.58% of the mitogenome and 29.28% of the plastome. Among them, the longest was 9,500 bp, and 13 fragments exceeded 500 bp. They were numbered by length, with each starting with ‘MTPT’. Eighteen complete genes and some intergenic regions were found on 52 homologous fragments. The homologous sequences included six plastidial PCGs (*rpl*23, *ycf*2, *rpo*C2, two *rps*7 and *rbc*L) and twelve tRNA genes (*trn*D-GUC, *trn*G-GCC, *trn*H-GUG, *trn*I-CAU, two *trn*M-CAU, four *trn*N-GUU, *trn*P-UGG, and *trn*S-UGA) (Table S6). In addition, some plastid gene fragments were found in the mitogenome (Table S5). There was no annotation on the homologous fragments MTPT14, MTPT19, MTPT24, and MTPT42. Detailed information about transferred genes was displayed (Table S5). The analysis detected 828 homologous fragments larger than 30 bp in *R. idaeus* between the nuclear genome and the mitogenome (Fig. 5B; Table S6). Among them, the longest was 3,593 bp, and ten fragments exceeded 700 bp. The total insert length is 115,610, accounting for 26.3% of the mitogenome. Only one complete *trn*M-CAU gene was annotated in NUMT3. Most homologous sequences were distributed on the sequence region between *rpl*10 and *trn*S-UGA (Table S6).

### Evolution and sequence collinearity

**Fig. 6:**
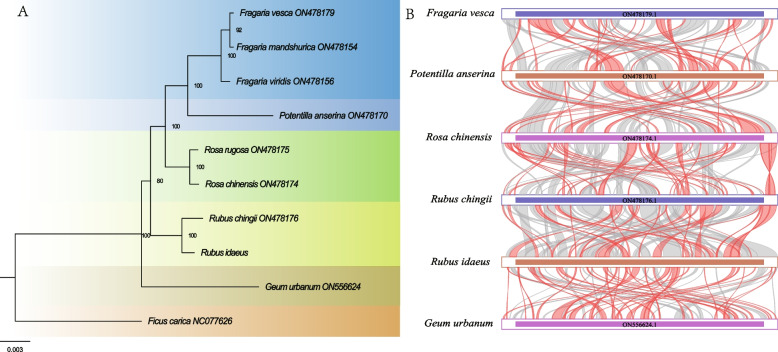
Phylogenetic analysis and homologous fragments. **A** Phylogenetic relationships of *R. idaeus* and closely related species. **B** Inter-species collinearity analysis. The bars represent the mitogenomes, and the ribbons display the homologous sequences between the adjacent species. The red areas indicate where the reversal occurred, and the gray areas represent regions of good homology

To further explore the evolutionary relationships in *R. idaeus*, a phylogenetic analysis of a few genera within Rosoideae with their mitogenomes available was conducted (Table S7). We used a shared conserved PCGs tree-building approach. A total of 25 PCGs (*atp*1, *atp*4, *atp*6, *atp*8, *atp*9, *ccm*B, *ccm*C, *ccm*Fc, *ccm*Fn, *cox*1, *cox*2, *cox*3, cob, *mat*R, *mtt*B, *nad1*, *nad*2, *nad*3, *nad*4, *nad*4L, *nad*5, *nad*6, *nad*7, *nad*9 and *rps*1) were used for phylogenetic analysis. The two *Rubus* species clustered in a single clade. All bootstrap values exceeded 0.8 in Rosoideae (Fig. 6A). The homologous regions between *R. idaeus* and the other four species (*Fragaria vesca, Potentilla anserina, Rosa chinensis,* and *Rubus chingii*) in Rosoideae were identified. The ribbon connecting the two genomes represents highly homologous collinear sequences with lengths larger than 500 bp. The largest collinear sequence spanned 31,476 bp, followed by 27,768 bp and 14,228 bp (Table S8). The results implied that the collinear blocks were rearranged in a different order in different species (Fig. 6B).

## Discussion

### Characteristics of the mtDNA of *R. idaeus* and multiple recombination conformations

The structure of mitochondria is also diverse, including linear, circular, highly branched, and networked, even within a single individual, possibly due to the highly frequent recombination [28–30]. The mitogenome of *R. idaeus* displayed a typical circular structure here (Fig. 1), which is same as most species of Rosaceae [17], implying that the length and structure of the small genome may have undergone a convergent evolution in history. The mitogenomes of angiosperms are in a state of dynamic change [31]. Although multiple recombinations have been detected, the big circle is the dominant conformation (Fig. 4). The mitogenome length is always influenced by the accumulation of different repetitive sequences and the incorporation of foreign sequences through horizontal or intracellular transfer [32, 33]. The length of the raspberry mitogenome was 438,947 bp, which is similar to that of *R. chingii* (472,138 bp) [34], displaying a medium length in the Rosaceae family (277.76 kb–535.73 kb). The repeat sequences and migration sequences contributed to the length of the raspberry. GC content is an important factor in assessing species. Raspberry was detected with a GC content of 44.1%, which is similar to species in Rosaceae (43.31%—45.62%) [34], indicating that GC content was very conservative during evolution for Rosoideae species.

A large number of repetitive sequences in plants often appear, including simple sequence repeats (SSRs), tandem repeats, and dispersed repeats [35–38]. SSRs, also known as microsatellites, consist of tandem repetitions of short oligonucleotide sequences. Due to their high polymorphism, SSRs are valuable tools in species identification and genetic diversity analysis, [39]. The analysis detected 52 SSRs from the mitogenome of raspberry (Fig. 3; Table S4). These SSRs serve as abundant molecular markers that can be used to assess germplasm diversity and facilitate species identification. The high A/T composition in SSRs may contribute to the overall AT richness observed in the mtDNA of *R. idaeus*. Among the dispersed repeats, the longest palindromic repeat included *the atp*1 gene (Table S6). Two copies of the *atp*1 gene in the mitogenome were identified, suggesting that repeated sequences may result in multiple copies of the genes.

Many studies demonstrated that repeat sequences have the potential effect to mediate the recombination of mitogenome [37, 38, 40]. In the mitogenome of *Gelsemium elegans,* four pairs of repeats were verified to mediate the homologous recombination into one major conformation and five minor conformations [40]. Similarly, the inverted repeats in *Taraxacum mongolicum* mitogenome meditated and resulted in two conformations, and two direct repeats broke one large circular molecule into two sub-genomic circular molecules [37]. Our study demonstrated plenty of repeated sequences (Fig. 3), showing that recombination may have occurred frequently in the mitogenome of red raspberry. This study finally identified and validated three pairs of repetitive sequences that meditated the major conformation and seven minor ones (Fig. 4). Different types of repeats can mediate different recombination (Fig. 4). In addition to the single circular conformation, multiple potential conformations mediated by repetitive sequences exist in raspberry (Fig. 4). These sequences and conformations may have changed dynamically in the mitogenomes. Our study further supported the presence of multiple conformations may exist in the mitogenomes of plants and revealed the way in which repeat sequences mediate homologous conformations. In addition, the formation of multiple mitochondrial genome conformations in plants may play a critical role in increasing genetic diversity and promoting adaptability.

### Codon usage of PCG analysis

Analysis of codon usage preference is important for studying species origin and evolution [41] For all the PCGs, ATG is the common start codon in plants, and UAA, UAG, and UGA are the common stop codons [42]. In *R. idaeus*, among the PCGs, the *nad*4L gene used the ACG as the start codon, and the rps4 gene used the TTG as the start codon. The same observation has been reported in other plants, such as *Primulina hunanensis, Medicago truncatula,* and *Brassica napus* (rapeseed) [43–45]. This may be caused by RNA editing modification*.* RSCU values surpassing 1.00 revealed a notable adenine/thymine (A/T) richness at the third codon position in *R. idaeus* mitogenome. The prevalence of NNA and NNT codons was similar to many other species, such as *Punica granatum*, *Cymbidium ensifolium*, *Amorphophallus albus* [29, 38, 46]. The pronounced bias towards AT nucleotides at the third codon may be a common pattern in the mitogenomes of land plants.

### Homology analysis

Extensively genetic material exchange between organelle genomes can be detected between organelle genomes in angiosperms [26, 35–37]. These include DNA sequences transfer from chloroplast to mitochondria (MTPTs) and sequences from nuclear to mitochondria (NUMTs). MTPTs typically constitute 1–12% of total mtDNA length [47]. This research revealed 46,456 bp MTPTs and 115,610 NUMTs in *R. idaeus*, accounting for approximately 10.58% and 26.3% of the whole mitogenome. Our research showed that gene transfer may have happened between the plastome and mitogenome, as well as between the mitogenome and nuclear genome. Compared with previous researches, we found homologous fragments vary among different species. Our annotation of the mitogenome of red raspberry demonstrated that certain fragments originated from the plastome (Fig. 5; Table S5). The plastome provides abundant non-native sequences for the mitogenome. The complete genes may remain functional in the mitogenome. Conversely, we speculated that the transferred gene fragments may have in greater or less experienced sequence loss. The formation of pseudogenes may be due to recombination after the sequence has been transferred to the mitochondria. In our analysis, a total of 12 complete tRNA were detected in homologous sequences, accounting for 46% (12/26) of the mitochondrial RNA genes (Fig. 5; Table S5). Compared with the PCGs, tRNA genes were more conservative in the mitogenome, suggesting that the tRNA genes play an indispensable role in mitochondria. Our study showed that gene transfer may have played an important role in plant evolution, and the fragments increased the diversity of the mitogenome of *R. idaeus*.

### Phylogenetic analysis and collinearity

The phylogenetic analysis based on lots of mitochondrial genes was conducted. The phylogenetic topology was highly consistent with previous studies inferred from chloroplast or nuclear data, indicating the important role of mitogenomes in constructing phylogenetic relationships. Homology analysis is crucial to elucidating the evolution of species [48]. Our study detected numerous homologous collinear blocks between *R. idaeus* and four species within the Rosoideae (Fig. 6). The mitogenome sequences of the five species in Rosoideae were extremely non-conservative in order, indicating that they may have experienced extremely frequent genome recombination in the long evolution process. Previous research also revealed the rearrangement and domestication as drivers of Rosaceae mitogenome plasticity [17]. Our research further sheds light on the frequent genome recombination, which may be a potential driver of mitogenome evolution.

## Methods

### Plant materials, DNA extraction and sequencing

The samples of *R. idaeus* were collected in Heicha Mountain in northwestern Shanxi Province (Longitude: 111.46222, Latitude: 38.39666). The specimen was deposited in the Wuhan Botanical Garden with the accession number HSM-2023fu. We sampled approximately five young leaves from the branch tips, collected them with liquid nitrogen in the field, and then transported them to the laboratory for storage in a −80°C freezer. The genomic DNA was extracted with modified CTAB methods [49]. Both Illumina and Oxford Nanopore data were sequenced. The short-paired reads were sequenced by Illumina HiSeq X (Illumina, Inc.; San Diego, CA, USA). For Oxford Nanopore sequencing, a library with an insert size of 10 kb was taken and sequenced by Nanopore PromethION sequencer (Oxford Nanopore Technologies, Oxford, UK). NanoPack was used to evaluate and control the quality of original reads [50].

### Genome assembly, polish and annotation

We assembled the mitogenome of *R. idaeus* with a hybrid strategy, which combined Illumina short reads with Nanopore long sequences. First, we used the SMARTdenovo software v3.0 [51] to do a de-novo assembly. We filtered the predicted mitochondrial contigs with the mitogenome reference of *Rubus chingii* (Accession number: NC065238). The selected contigs were polished with Miniasm V0.3 + Racon V1.5.0 for three iterations [52, 53]. We filtered the mitochondrial reads from Illumina short reads data according to mapping the clean short reads to the predicted mitochondrial contigs with bowtie2 and SAMtools [54, 55]. Finally, we used Unicycler v0.5.0 [56] to assemble the mitogenome combined with the filtered Illumina short reads and Nanopore long reads. The GFA format files generated by Unicycler were visualized using Bandage [57].

We annotated the mitogenome of *R. idaeus* using the Geseq webserver (https://chlorobox.mpimp-golm.mpg.de/geseq.html) [58] with the reference genome of *Rubus chingii* (Accession number: NC065238), the tRNAs and rRNAs were identified using tRNAscan-SE [59]. We drew the genome circular map of the mitogenome with OGdraw [60], and all the annotations of the mitogenome were reviewed carefully and manually corrected with Apollo software [61].

### Codon preference and repeat element analysis

We extracted the PCGs of the genome using Phylosuite (Zhang et al., 2020). The PCGs of the mitogenome were analyzed for codon preference using MEGA X [62], and relative synonymous codon usage (RSCU) values were calculated [63]. We detected three kinds of repetitive sequences, including simple sequence repeats (SSRs), tandem, and dispersed repeats. SSRs were detected using the online website MISA (https://webblast.ipk-gatersleben.de/misa/) [64]. We set minimum numbers of mono-, di-, tri-, tetra-, penta-, and hexanucleotides as 10, 5, 4, 3, 3, and 3, respectively. We detected the forward, reverse, palindromic, and complementary repeat sequences using online REPuter (https://bibiserv.cebitec.uni-bielefeld.de/reputer/) [65] with a Hamming distance of three, minimal repeat size of 30, and maximum computed repeats of 5000. We detected the tandem repeats using the online Tandem Repeats Finder (TRF) ( http://tandem.bu.edu/trf/trf.html) [65]. The results of repeat element analysis were visualized using the Circos package which was implemented in TBtools [66].

### Identification and validation of repeat-mediated homologous recombination

We detected repetitive sequences that might mediate recombination using BLASTN [67] with an e-value cut-off of 1e-5. We extracted each pair of repetitive sequences longer than 200 bp as candidate recombination sequences (CRS), along with their 1000 bp flanking regions. The mitogenome displayed two types of recombination for completely identical repeat sequences and six types of recombination for incompletely identical repeat sequences (Fig. S1). We aligned all CRSs to the corrected ONT reads, and results with over 95% similarity were considered homologous recombination sequences. PCR amplification was conducted using designed primers (Table S9) to validate the recombination. We conducted PCR experiments using 1 µl DNA, 1 µl 10 µM each pair of primers, 13 μrimers, 13 µl PCR mix. The PCR cycling conditions were as follows: initial denaturation at 94°C for 3 min 30 cycles of 94°C for 30 s, 54–60°C for 30 s and 72° and 72of 9, 72°72nd 72o min. PCR products were sequenced using the Sanger method.

### Mitochondrial plastid DNAs (MTPTs) and nuclear mitochondrial DNA segments (NUMTs)

The mitogenome of plants often contains extensive sequences that have migrated from plastomes and nuclear genomes [26, 35–37]. In this study, we assembled the mitogenome of *R. idaeus* and downloaded the plastome of *R. idaeus* (OR698909). We also downloaded the nuclear genome of the *R. idaeu* cultivar ‘Autumn Bliss’ from NCBI with the Bioproject ID: PRJNA886864 [12]. The nuclear genome of *R. idaeus* was 263 Mb and contained seven chromosomes. First, we compared the mitogenome and plastome sequences using BASTN [67] with an e-value cut-off of 1e-5 to identify MTPTs. The NUMTs were identified with BLASTN using the same method and parameters. Then, the distribution of MTPTs and NUMTs across all the genomes was visualized using the Circos [68] package implemented in TBtools [66]. (Fig. 5a).

### Phylogenetic analysis and collinear analysis in Rosoideae

We downloaded eight mitogenomes of Rosoideae species and one outgroup from NCBI (https://www.ncbi.nlm.nih.gov/) for phylogenetic analysis. We set *Ficus carica* (accession number: NC077626) from Moraceae as outgroups, as Moraceae and Rosaceae are the most phylogenetically related among the species whose mitogenomes have been published. The mitogenomes were downloaded with the following accession numbers: *Fragaria mandshurica* (ON478154), *Fragaria viridis* (ON478156), *Potentilla anserina* (ON478170), *Rosa chinensis* (ON478174), *Rosa rugosa* (ON478175), *Rubus chingii* (ON478176), *Fragaria vesca* (ON478179), *Geum urbanum* (ON556624). Shared genes of mitogenomes were filtered, extracted, and concatenated using PhyloSuite software [62]. Multiple sequence alignment was conducted using MAFFT [69]. Raxml-HPC-AVX was used for the phylogenetic analysis with the GTRGAMMA model [70]. Finally, the maximum-likelihood tree was visualized using FigTree v1.4.2 [71].

We selected five additional species from five genera in Rosoideae for collinear analysis [17]. The five species included *Fragaria vesca*, *Potentilla anserina*, *Rosa chinensis*, *Rubus chingii*, and *Geum urbanum*. We identified the collinear blocks in sequence using BLASTn [67]. Collinear blocks longer than 500 bp were selected for subsequent analysis. Multiple synteny plots were drawn using the MCscan source program [72], which is implemented in TBtools [66].

## Conclusion

In this study, we sequenced and assembled the mitogenome of *R. idaeus* by integrating Illumina short reads with Nanopore long reads. The popular fruit species displayed a typical circular structure with a total length of 438,947 bp. We annotated 33 PCGs, 26 tRNA, and three rRNA genes in the mitogenome. Additionally, comprehensive analyses, including codon usage, repeated sequences, repeat-mediated genome recombination, chloroplast and nuclear to mitochondrion DNA transformation, and synteny, were conducted. Our study revealed the important role of long repeat sequences in mediating homologous recombination and indicated that gene transfer has facilitated the diversification of mitochondrial genomes in plants. Our research also provides valuable molecular resources for the introduction, conservation, and breeding of red raspberry in future studies.

## Supplementary Information

Supplementary Material 1. Fig. S1. Two types of recombination for completely identical repeat sequences and six types of recombination for incompletely identical repeat sequences.

Supplementary Material 2.

## Data Availability

The assembled organelle genome sequences have been deposited in NCBI with accession number PP003260. The sequencing reads used for mitogenome assembly in this study have been released on the NCBI with those accession numbers PRJNA1142221 (BioProject), SAMN42930120 (BioSample), and SRR30129797 (SRA). The sample was deposited in the Wuhan Botanical Garden with the accession number HSM-2023fu.
