# Supplementary material for: Assembly and comparative analysis of the complete mitochondrial genome of red raspberry (Rubus idaeus L.) revealing repeat-mediated recombination and gene transfer
Source: BMC Plant Biol. 2025 Jan 22;25:85. doi: 10.1186/s12870-024-05969-7 (PMC11752677; doi:10.1186/s12870-024-05969-7)
Supplement: Supplementary file 1 — Supplementary Material 1. Fig. S1. Two types of recombination for completely identical repeat sequences and six types of recombination for incompletely identical repeat sequences. [file 12870_2024_5969_MOESM1_ESM.pdf]

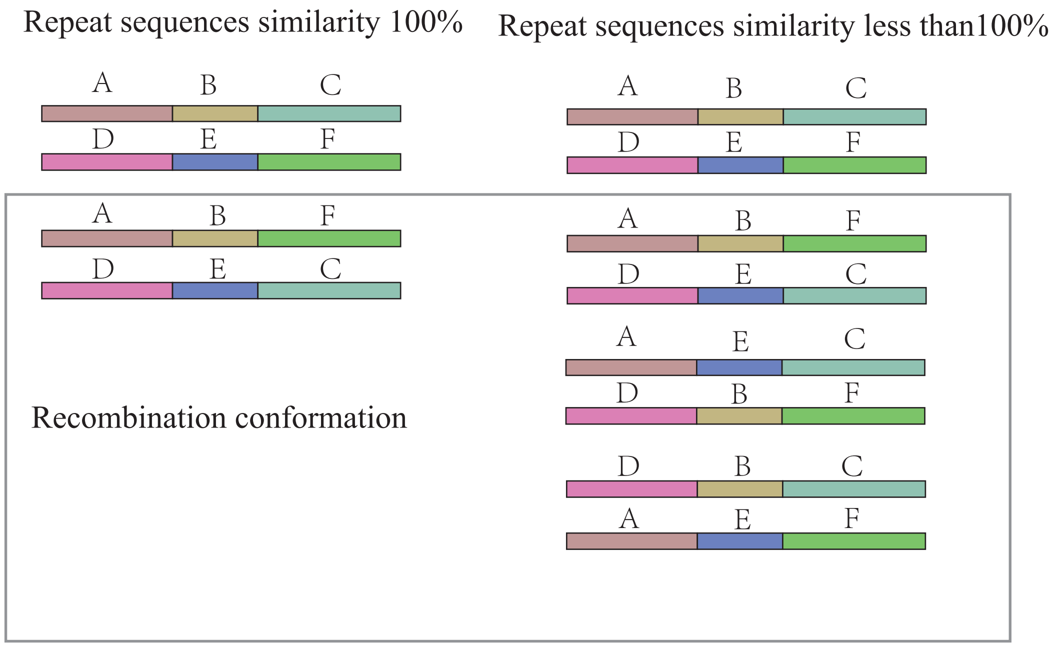
Repeat sequences similarity 100%
Repeat sequences similarity less than100%
A B C
D E F
A B C
D E F
A B F
D E C
A B F
D E C
A E C
D B F
Recombination conformation
D B C
A E F
